# Sterol biosynthesis regulates TLR signaling and the innate immune response in a Smith-Lemli-Opitz syndrome model

**DOI:** 10.1172/JCI167633

**Published:** 2024-01-18

**Authors:** Kristin Gabor, Emily V. Mesev, Jennifer Madenspacher, Julie Meacham, Prashant Rai, Sookjin Moon, Christopher A. Wassif, Saame Raza Shaikh, C.J. Tucker, Peer Karmaus, Simona Bianconi, Forbes D. Porter, Michael B. Fessler

**Affiliations:** 1 Immunity, Inflammation and Disease Laboratory, National Institute of Environmental Health Sciences, NIH, Research Triangle Park, North Carolina, USA.; 2 Section on Molecular Dysmorphology, Eunice Kennedy Shriver National Institute of Child Health and Human Development, NIH, Bethesda, Maryland, USA.; 3 Department of Nutrition, Gillings School of Global Public Health and School of Medicine, University of North Carolina at Chapel Hill, Chapel Hill, North Carolina, USA.; 4 Fluorescence Microscopy and Imaging Center, National Institute of Environmental Health Sciences, NIH, Research Triangle Park, North Carolina, USA.

**Keywords:** Immunology, Inflammation, Cholesterol, Innate immunity, Lipid rafts

**To the Editor:** TLRs are activated in cholesterol-enriched lipid raft membrane microdomains (1). Depleting raft cholesterol attenuates proinflammatory signaling by TLR4, the receptor for bacterial LPS. However, raft cholesterol has generally been manipulated using pharmacologic strategies with limited selectivity and uncertain physiological relevance (1).

Smith-Lemli-Opitz syndrome (SLOS; OMIM 270400) is an autosomal recessive, multiple malformation, neurodevelopmental disorder caused by pathological variants of 7-dehydrocholesterol reductase (*DHCR7*), a terminal enzyme in cholesterol biosynthesis (2). The most common of 8 described inborn errors of cholesterol synthesis, SLOS has a carrier frequency of approximately 1%–2% in Northern Europeans, with lower rates in people of other races (2). Increased susceptibility to infection has been described, but it is poorly understood.

Rafts in *DHCR7*-mutant cells exhibit defects due to reduced cholesterol and increased 7-dehydrocholesterol content (3), although not all raft-dependent signaling is defective in SLOS cells (4). To test TLR signaling, we cultured dermal fibroblasts from individuals with SLOS with defined pathological *DHCR7* variants (Supplemental Table 1 and Supplemental Methods; supplemental material available online with this article; https://doi.org/10.1172/JCI167633DS1). SLOS fibroblasts exhibited elevated 7-dehydrocholesterol and reduced cholesterol, as expected (Supplemental Figure 1A). Upon LPS stimulation, SLOS fibroblasts produced less IL-6 and IL-8 than controls (Figure 1A). Rank ordering of the patients by clinical severity score revealed an inverse relationship between clinical severity and LPS-induced IL-6 production but not IL-8 production (Figure 1B and Supplemental Figure 1B). Whole blood from individuals with SLOS stimulated ex vivo with LPS also induced lower cytokine levels compared with control blood; the patients with the lowest serum cholesterol tended to have the lowest levels of cytokine induction (Figure 1C, Supplemental Figure 1C, and Supplemental Table 2). Compared with WT macrophages, macrophages from *Dhcr7*-hypomorphic (p.T93M/Δ) mice (5) also produced reduced cytokine protein in response to LPS and TLR2 ligands (Pam3CSK4, *L*. *monocytogenes*) but had normal responses to poly(I:C) (TLR3 ligand) and TNF-α (Figure 1D and Supplemental Figure 1D). *Il6* mRNA induction was also attenuated, whereas *Tnf* and *Il1b* mRNA was augmented, suggesting differential posttranscriptional regulation (Supplemental Figure 1E).

RAW 264.7 macrophages treated with the DHCR7 inhibitor BM15.766 (5), under lipid-depleted conditions to model SLOS (Supplemental Figure 1F), displayed attenuated induction of several myeloid differentiation primary response 88–dependent (MyD88-dependent) and MyD88-independent cytokines/chemokines representative of the two major adaptor pathways downstream of TLR4 (Supplemental Figure 2, A and B). Cell staining with Alexa Fluor 488–conjugated cholera toxin subunit B (CtB), a probe for raft gangliosides, revealed reduced signal intensity, foci, and clustering, suggesting reduced raft mass, number, and coalescence, respectively (Figure 1, E–G, and Supplemental Figure 2, C and D). The raft cholesterol probe fPEG-cholesterol (1) also indicated reduced signal intensity (Figure 1H), as did ALOD4, a probe for accessible membrane cholesterol (1) (Supplemental Figure 2E). *Dhcr7*^T93M/Δ^ murine macrophages did not display a consistent reduction in CtB or fPEG-cholesterol signal (data not shown) but exhibited reduced ALOD4 signal (Supplemental Figure 2F).

TLR4 ligation by LPS induces recruitment of MyD88 to rafts (1), where it interacts with IL-1 receptor–associated kinase 2 (IRAK2) and TNF receptor–associated factor 6 (TRAF6) to form the “Myddosome” complex, activating the kinase JNK. TLR4 surface display was reduced in SLOS model RAW 264.7 macrophages (Supplemental Figure 2G and Figure 1I). LPS recruited IRAK2 and TRAF6 to MyD88 in control macrophages, but negligible Myddosome assembly was detected in SLOS-like macrophages (Figure 1J). JNK activation was also attenuated, as was p38 activation to a more variable extent (Supplemental Figure 2H).

In response to intratracheal *Klebsiella pneumoniae*, *Dhcr7*^T93M/Δ^ mice exhibited reduced airspace neutrophilia (Figure 1K), denoting a defective innate immune response. *Dhcr7*^T93M/+^ mice exhibited an intermediate phenotype. Despite attenuated neutrophilia, no change in pathogen clearance or serum cytokines was detected (Supplemental Figure 2, I and J). In response to influenza A virus, *Dhcr7*^T93M/Δ^ lungs exhibited attenuated induction of interferon-stimulated genes and cytokines; lower *Ly6g*, denoting reduced neutrophil infiltration; and increased expression of the viral PA gene, suggesting higher viral burden (Figure 1L and Supplemental Figure 2K).

The major implications of our findings are 3-fold. First, SLOS provides genetic evidence for the requirement for sterol biosynthesis in innate immunity. Second, our findings raise the possibility that compromised TLR signaling may underlie susceptibility to infection in SLOS. Third, as several widely used medications (e.g., trazodone, aripiprazole) inhibit DHCR7, with more marked effects in *DHCR7* mutation carriers (6), we propose that patients on these medications may have suppressed TLR responses and that DHCR7 inhibitors should be examined for repurposing as modulators of innate immune overactivation. Future studies are warranted to comprehensively characterize plasma membrane structure in SLOS and to define whether cholesterol deficiency and/or 7-dehydrocholesterol excess impair TLR signaling.

## Supplementary Material

Supplemental data

Unedited blot and gel images

Supporting data values

## Figures and Tables

**Figure 1:**
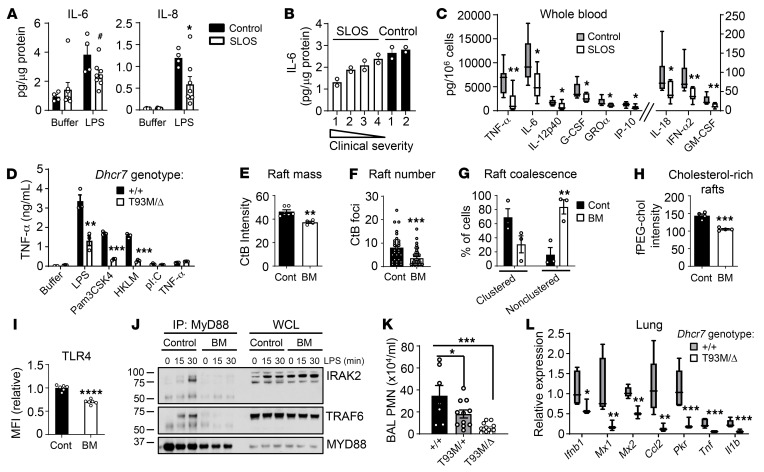
Attenuated TLR signaling in SLOS. (**A**) Fibroblasts from individuals acting as controls (*n* = 2) and patients with SLOS (*n* = 4) were stimulated, and media were assayed for cytokines. (**B**) Fibroblast IL-6 production rank ordered by clinical severity score. (**C**) Whole blood was LPS stimulated and assayed for cytokines (*n* = 7–11). (**D**) Peritoneal macrophages from mice of indicated genotypes were stimulated (HKLM, heat-killed *L*. *monocytogenes*) and TNF-α in media was quantified. (**E**–**I**) RAW 264.7 macrophages were treated with vehicle or BM15.766 (BM), stained with Alexa Fluor 488–cholera toxin subunit B (CtB), and imaged for (**E**) total intensity, (**F**) CtB^+^ foci/cell, and (**G**) clustering of foci. (**H** and **I**) Signal for (**H**) fPEG-cholesterol and (**I**) anti-TLR4 antibody was quantified. (**J**) Macrophages pretreated with vehicle/BM were LPS stimulated. MyD88 immunoprecipitates (IP) and whole cell lysates (WCL) were immunoblotted for indicated targets. (**K**) Bronchoalveolar lavage neutrophils (PMN) quantified 24 hours after *K*. *pneumoniae* lung infection in mice of indicated genotypes (*n* = 7–12). (**L**) qPCR for indicated targets in mouse lungs 3 days after infection with influenza A virus (*n* = 3–5). Data represent mean ± SEM and are representative of 2–3 independent experiments. In **C** and **L**, boxes depict the 25th–75th percentile around median; whiskers represent the minimum and maximum. ^#^*P* = 0.05; *P < 0.05; **P < 0.01; ***P < 0.001 (unpaired 2-tailed *t* test for all except 1-way ANOVA with Dunnett’s post test for **K**).

## Version 1 01/18/2024

In-Press Preview

## Version 2 03/15/2024

Electronic publication
