# Supplementary material for: Sterol biosynthesis regulates TLR signaling and the innate immune response in a Smith-Lemli-Opitz syndrome model
Source: J Clin Invest. 2024 Jan 18;134(6):e167633. doi: 10.1172/JCI167633 (PMC10940081; doi:10.1172/JCI167633)
Supplement: Supplemental data [file jci-134-167633-s043.pdf]

Sterol biosynthesis regulates TLR signaling and the innate immune response in Smith-Lemli-Opitz syndrome model

Kristin Gabor[1], Emily V. Mesev[1], Jennifer H. Madenspacher[1], Julie Meacham[1], Prashant Rai[1], Sookjin Moon[1], Christopher A. Wassif[2], Saame Raza Shaikh[3], C.J. Tucker[4], Peer Karmaus[1], Simona Bianconi[2], Forbes D. Porter[2], Michael B. Fessler[1]

[1]Immunity, Inflammation and Disease Laboratory and [4]Fluorescence Microscopy and Imaging Center, National Institute of Environmental Health Sciences, NIH, Research Triangle Park, NC 27709
[2]Section on Molecular Dysmorphology, *Eunice Kennedy Shriver* National Institute of Child Health and Human Development, NIH, Bethesda, MD 20892
[3]Department of Nutrition, Gillings School of Global Public Health and School of Medicine, University of North Carolina at Chapel Hill, Chapel Hill, NC 27599

Supplemental Material

Methods

Sex as a biological variable. Our study examined male and female humans and mice, and similar findings were noted for both sexes.

Reagents. BM15.766 sulfate, penicillin, and streptomycin were from Sigma (St Louis, MO). DMEM and fetal bovine serum were from American Type Culture Collection (Rockville, MD). Lipoprotein-depleted sera (LPDS) was from Kalen Biomedical, LLC (Montgomery Village, MD). His-tagged domain 4 of anthrolysin O (ALOD4), a gift from Arun Radhakrishnan (Addgene plasmid # 111026 ; http://n2t.net/addgene:111026 ; RRID:Addgene_111026)(1), was expressed and purified as previously described (2).

Mice. *Dhcr7* $^{T93M/\Delta}$ mice have been previously reported (3). All experiments used sex-matched littermate controls of age ~6-9 weeks. All experiments were performed in accordance with the Animal Welfare Act and the US Public Health Service Policy on Humane Care and Use of Laboratory Animals after review by the Animal Care and Use Committee of the NIEHS.

Mouse peritoneal macrophages. Peritoneal exudate macrophages were isolated using standard procedures (4). Briefly, macrophages were harvested by peritoneal lavage 96h after i.p. Brewer's thioglycollate (2 ml, 4%) and then plated for 72h in either complete (DMEM/10% FBS) or lipid-depleted (DMEM/7.5% LPDS) media before stimulation. Macrophages were stimulated for 2h with vehicle (1x PBS), Pam3CSK4 (Invivogen, San Diego, CA; 100 ng/ml), LPS (List Biologicals, Campbell, CA; 10 ng/ml), poly(I:C) (Invivogen, San Diego, CA) or heat-killed *L. monocytogenes* (HKLM; Invivogen, San Diego, CA; 10^8 cells/ml), or TNFα (R&D Systems; 10 ng/mL).

Cell culture. Skin fibroblasts were obtained from individuals with SLOS and confirmed by both biochemical (sterol) testing and *DHCR7* genotyping in accordance with an NICHD IRB-approved

protocol and informed consent, as reported (5). Control fibroblasts were obtained from the ATCC. Fibroblasts were propagated (37°C, 5% CO_2) in DMEM supplemented with 10% FBS. Prior to study, fibroblasts were grown for 4-5 days in McCoy's 5A medium supplemented with 7.5% LPDS as reported (5). RAW 264.7 macrophages were treated for 4-5 days with 0.1% DMSO vehicle in DMEM/10% FBS, or with 5 μM BM15.766 in DMEM/7.5% LPDS, and then were all washed and changed to DMEM/7.5% LPDS prior to LPS stimulation. All media were supplemented with 0.20 mg/ml L-glutamine, 100 U/ml penicillin G sodium and 100 mg/ml streptomycin sulfate.

Confirmation of SLOS biochemical phenotype. Cell cholesterol and 7DHC was confirmed by GC/MS as reported (5). Neutral sterols were extracted 1:1 with ethyl acetate and taken to dryness under a stream of nitrogen before derivation with BSTFA +1% TMCS (Thermo Fisher Scientific). Samples were loaded on a Trace Gas Chromatogram (Thermo Electron) utilizing a ZB 1701 30m X 0.25mm X 0.25um (Phenomenex) column for separation and analyzed in full scan mode on a single quadrupole mass spectrometer (Thermo Electron). All GC/MS data was analyzed using the Excalibur software package normalized to the internal surrogate standard coprostanol.

Murine pneumonia model. Mice were administered 2000 CFU *K. pneumoniae* (ATCC 43816) by oropharyngeal aspiration as previously reported (6). BALF was collected as described (4) and cells resuspended in PBS, cytospun (Thermo Scientific) onto glass slides, and stained with PROTOCOL™ Hema 3 Fixative and Solutions (Fisher Scientific, Kalamazoo, MI) for manual cell differential counting. Bacterial burden was quantified in lung homogenates and peripheral blood using serial dilution (6, 7). Alternatively, mice received ~20 ffu of Influenza A/PR8/34 virus via oropharyngeal aspiration and lungs were necropsied 3d post-infection for qPCR analysis.

Cytokine analysis. Cytokines were quantified by ELISA (BioLegend) or by Bioplex assay (Bio-Rad). In analysis of human whole blood, cytokine levels were normalized to the sum of absolute

monocyte plus neutrophil count for each subject, as determined by complete blood count/differential drawn at the same time.

RNA isolation and RT-qPCR. Total RNA was isolated from cells and tissues by RNEasy kit (Qiagen) following manufacturer's instructions. cDNA was generated from 1.0 µg of purified RNA using TaqMan reverse transcription reagents from Applied Biosystems (Foster City, CA). Real-time PCR was performed with Taqman PCR Mix (Applied Biosystems) in the HT7900 ABI sequence Detection System (Applied Biosystems). Predesigned TaqMan primers were purchased from Applied Biosystems. Gene expression levels were normalized to GAPDH or 18S as indicated and expression levels in untreated control samples were set as a value of 1.0.

Fluorescence microscopy. GM1 was stained using Alexa Fluor 488- or 594-Cholera Toxin B (CtB) (Life Technologies/Molecular Probes) as reported (8, 9). Cells in growth medium were incubated in AF-CtB (1 µg/ml, 10 min, 4°C), washed, and cross-linked with anti-CtB antibody provided by the manufacturer (200-fold dilution, 15 min, 4°C). In other studies, cells were stained with fPEG-cholesterol, as previously described (10). Cells were fixed in chilled 1x PBS containing 4% paraformaldehyde (15 min, RT), and then washed several times with 1x PBS and mounted with ProLong Antifade Gold Kit (Molecular Probes). Imaging was performed with a Zeiss LSM 880 microscope with a 63x objective/1.30 oil and ZEN software. For AF594-CtB, an Alexa red filter set was used with a 458/561 dichroic beam splitter and a 561 nm laser was used. For analysis of raft population (clustered vs. non-clustered), a minimum of 12 cells per treatment from 3 individual experiments were analyzed in a blinded manner following equal background subtraction using ImageJ software. A previously published method was used to assign cells into one of two categories (11). Clustered cells exhibited a punctate pattern of staining whereas non-clustered cells showed a uniform distribution of staining. If a cell displayed a mixture of clustered and non-clustered domains, it was classified as clustered. Raft clusters were also analyzed using dSTORM

analysis. Specifically, RAW 264.7 cells were cultured for 4 days in complete media/vehicle or lipoprotein-deficient media/BM15.766 (5 μM) on a No. 1.5 dish (Mattek; Cat. # P35G-1.5-14-C), washed twice with PBS, and incubated in a 1mg/mL CtB conjugated to Alexa Fluor-647 (CtB-647) in PBS (4°C, 10 minutes). Cells were washed twice in PBS, fixed in 4% formaldehyde (RT, 15 minutes), and washed again. A coverslip was then placed on top of the well, and a mercaptoethylamine-based STORM ABC solution [Solution A (30mM Tris/Cl pH 8.5; 1mM EDTA; 6.25 uM glucose oxidate; 2.5 uM catalase), solution B (250 mM cysteamine-HCL) and solution C (250 mM glucose) mixed at a 8:1:1 ratio] was injected into the well. 3D super-resolution images were captured on an Ando Dragonfly 505 microscope (10,000 images in burst mode; 637nm laser at 100%). Clusters of CtB-647 fluorescence >200nm in length were identified and counted for each cell imaged using Huygens localizer software. In other experiments, cells were washed with PBS, treated with His-ALOD4, washed again, and then treated with Alexa Fluor 488-conjugated anti-His antibody (R&D systems IC050G) after initially being left untreated or treated with mβCD (5 mM, 30 minutes) (Sigma); cells were then imaged with a Zeiss LSM 880 (63x objective) with Airyscan.

Flow cytometry. Cells were incubated in blocking buffer with 5% mouse serum, 5% rat serum, and anti-mouse CD16/32 (clone 2.4G2, Biolegend), and then stained with anti-TLR4 antibody (SA15-21; Biolegend #145405) or isotype control antibody. The cells were then washed, resuspended, and analyzed on a LSR-II Flow Cytometer (BD Biosciences) using FACSDiva (BD Biosciences) and FlowJo software (Tree Star).

Immunoprecipitation and immunoblotting. Standard methods were used for immunoprecipitation (IP) and immunoblotting (8). Goat anti-MyD88 (R&D Systems) and Protein G-Sepharose were used for IP. An equal protein mass (BCA assay) was resolved on 4–20%

Tris-HCl gels (BioRad), transferred to nitrocellulose (Amersham Biosciences), and probed with primary antibodies, including goat anti-MyD88 (1:1000; R&D Systems), rabbit anti-TRAF6 (1:1000; Santa Cruz), rabbit anti-IRAK2 (1:1000; ProSci), rabbit anti-PO_4-p38 (1:500; Cell Signaling Technology), rabbit anti-PO_4-JNK (1:500, Cell Signaling Technology), and rabbit anti-p38 (1:500, Santa Cruz). Membranes were washed in Tween Tris buffered saline (TTBS), and exposed to species-specific HRP-conjugated secondary antibody (GE Healthcare; 1:5,000; 60 min) in 5% milk/TTBS. After further washes, signal was detected with HyGlo (Quick Spray) chemiluminescent HRP antibody detection reagent, followed by film exposure (HyBlot CL, Denville Scientific).

Whole blood stimulation. Blood was obtained from individuals with SLOS and healthy individuals using a 10 ml syringe containing sodium-heparin (50 IU/ml final concentration). Within 10 minutes of collection, 1 ml of whole blood was aliquoted into prewarmed TruCulture tubes (Myriad RBM) and incubated in a dry block incubator at 37°C for 24h (LPS, control). Tubes were then opened and cells sedimented. The serum supernatant was aliquoted and immediately frozen at -80°C until use.

Statistical analysis. Data were analyzed using the GraphPad Prism statistical software (San Diego, CA). Data are represented as mean $\pm$ SEM. Two-tailed Student's *t*-test was applied for comparisons of two groups and ANOVA with Dunnett's post-test for comparisons of more than two groups. For all analyses, $P<0.05$ was considered significant.

Study Approval. Individuals with SLOS and age-, gender-, race- and ethnicity-matched controls were enrolled in a study at the NIH Clinical Center (NCT00001721) and NIEHS Clinical Research Unit, respectively. Informed consent was obtained from all individual participants (or their guardians, as appropriate due to age and/or cognitive impairment) included in the study, prior to

study participation, following a protocol approved by the Institutional Review Board at both NICHD and NIEHS. All procedures performed in studies involving human participants were done in accordance with the ethical standards of the institutional review committee and with the 1964 declaration of Helsinki and comparable ethical standards. All experiments involving animals were performed in accordance with the Animal Welfare Act and the U.S. Public Health Service Policy on Humane Care and Use of Laboratory Animals after review by the NIEHS Animal Care and Use Committee.

Data availability. Values for all data points in graphs are reported in the Supporting Data Values file.

Table S1. SLOS participant cohort for fibroblast studies

Participant	Clinical Severity Score*	*DHCR7* mutation
1	56	c.964-1G>C/p.L109P
2	33	c.964-1G>C /p.T93M
3	28	p.T93M/p.W151X
4	11	c.964-1G>C /p.T93M

*Score <20 = mild; 20-35 = typical; >35 = severe [3].

Table S2. Cohort for *ex vivo* whole blood stimulation studies*

Participants	Age (mean [years])	Gender (Female, n [%])	Race	Medications
Control (n=12)	11.8	7 (58%)	11 W, 1 A	
1				None
2				Multivitamin
3				Multivitamin
4				None
5				None
6				None
7				Multivitamin
8				None
9				None
10				None
11				Multivitamin
12				None
SLOS (n=7)	11.6	4 (57%)	6 W, 1 A	
1				Norditropin flex pen 1.5 mg sc 6d/wk; Focalin 5 mg prn po in am 3-5 times/wk
2				Melatonin 5 mg gummies in pm, methylphenidate HCL tabs 5 mg and 27 mg extended release, guanfacine Hcl ER tabs 4 mg, desloratidine 5 mg, montelukast 5 mg, cholesterol cherry suspension (150 mg/ml) 30 ml per day
3				Cholesterol liquid suspension 150 mg/ml 18 ml/d, Miralax 2 teaspoons/d, Ibuprofen prn
4				Guanfacine 6-7 mg/d, Zyrtec 10 mg prn, Doxycycline 50 mg tablet twice/d,Seroquel 25 mg daily in pm
5				Cholesterol 150 mg/ml suspension 19.5 ml daily, Zyrtec 7 mg/d, Miralax 5 ml/d,Gapapentin 9 ml/d, Melatonin 5 ml/d, Zofran 3.75 ml prn
6				guanfacine 1 mg/d, multivitamin
7				none

*A = Asian; W = White. SLOS participant numbers do not correspond to those in Table S1.

References

1. Gay A, Rye D, and Radhakrishnan A. Switch-like responses of two cholesterol sensors do not require protein oligomerization in membranes. *Biophys J.* 2015;108(6):1459-69.
2. Endapally S, Infante RE, and Radhakrishnan A. Monitoring and Modulating Intracellular Cholesterol Trafficking Using ALOD4, a Cholesterol-Binding Protein. *Methods Mol Biol.* 2019;1949:153-63.
3. Correa-Cerro LS, Wassif CA, Kratz L, Miller GF, Munasinghe JP, Grinberg A, et al. Development and characterization of a hypomorphic Smith-Lemli-Opitz syndrome mouse model and efficacy of simvastatin therapy. *Hum Mol Genet.* 2006;15(6):839-51.
4. Madenspacher JH, Morrell ED, Gowdy KM, McDonald JG, Thompson BM, Muse G, et al. Cholesterol 25-hydroxylase promotes efferocytosis and resolution of lung inflammation. *JCI Insight.* 2020;5(11).
5. Wassif CA, Krakowiak PA, Wright BS, Gewandter JS, Sterner AL, Javitt N, et al. Residual cholesterol synthesis and simvastatin induction of cholesterol synthesis in Smith-Lemli-Opitz syndrome fibroblasts. *Mol Genet Metab.* 2005;85(2):96-107.
6. Lin WC, Gowdy KM, Madenspacher JH, Zemans RL, Yamamoto K, Lyons-Cohen M, et al. Epithelial membrane protein 2 governs transepithelial migration of neutrophils into the airspace. *J Clin Invest.* 2020;130(1):157-70.
7. Madenspacher JH, Azzam KM, Gowdy KM, Malcolm KC, Nick JA, Dixon D, et al. p53 Integrates host defense and cell fate during bacterial pneumonia. *J Exp Med.* 2013;210(5):891-904.
8. Aloor JJ, Azzam KM, Guardiola JJ, Gowdy KM, Madenspacher JH, Gabor KA, et al. Leucine-rich repeats and calponin homology containing 4 (Lrch4) regulates the innate immune response. *J Biol Chem.* 2019;294(6):1997-2008.
9. Gale SC, Gao L, Mikacenic C, Coyle SM, Rafaels N, Murray Dudenkov T, et al. APOepsilon4 is associated with enhanced in vivo innate immune responses in human subjects. *J Allergy Clin Immunol.* 2014;134(1):127-34.
10. Madenspacher JH, Draper DW, Smoak KA, Li H, Griffiths GL, Suratt BT, et al. Dyslipidemia induces opposing effects on intrapulmonary and extrapulmonary host defense through divergent TLR response phenotypes. *J Immunol.* 2010;185(3):1660-9.
11. Shaikh SR, Rockett BD, Salameh M, and Carraway K. Docosahexaenoic acid modifies the clustering and size of lipid rafts and the lateral organization and surface expression of MHC class I of EL4 cells. *J Nutr.* 2009;139(9):1632-9.

Figure S1

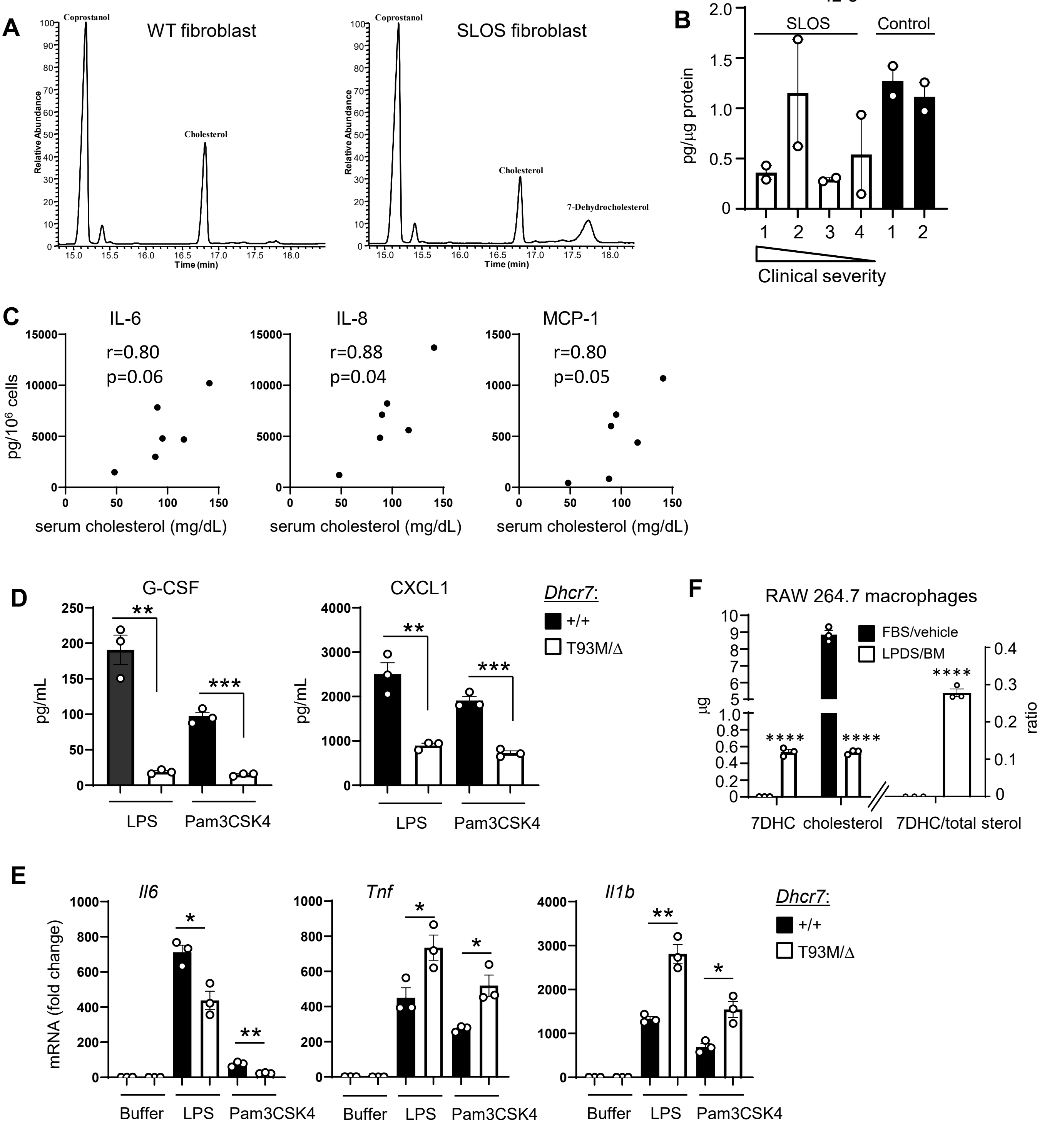

Figure S1. (**A**) Exemplary chromatograms of sterols (coprastanol [standard], cholesterol, 7-dehydrocholesterol) extracted from WT vs. SLOS patient dermal fibroblasts cultured for 5d in lipoprotein-deficient serum media. Compared to control, cholesterol is reduced and 7-dehydrocholesterol is detectable in the SLOS sample. (**B**) Dermal fibroblast IL-8 production by ELISA was rank-ordered by clinical severity score of individuals with SLOS (see Table S1). (**C**) Whole blood from individuals with SLOS (n=6) was stimulated *ex vivo* with LPS and cytokine release assayed. Cytokine levels were plotted against serum cholesterol of the individuals. Correlation r values and permutation-based p-values were determined, as shown. (**D**-**E**) Murine peritoneal macrophages of the indicated genotype were stimulated by LPS or Pam3CSK4 for 120 min and (D) the media (Bioplex of protein) or (E) cell lysate (qPCR of mRNA [normalized to +/+ vehicle]) assayed for the cytokines shown (n=3, representative of 2 independent experiments). (**F**) RAW 264.7 macrophages ($3x10^5$) were cultured in complete media with vehicle or lipoprotein-deficient serum (LPDS) media with BM15.766 (BM) for 5 days. Cell lipid extracts were then assayed by GC-MS for 7-DHC and cholesterol. *, p<0.05; **, P<0.01; ****, P<0.0001 by unpaired 2-tailed t-test.

Figure S2

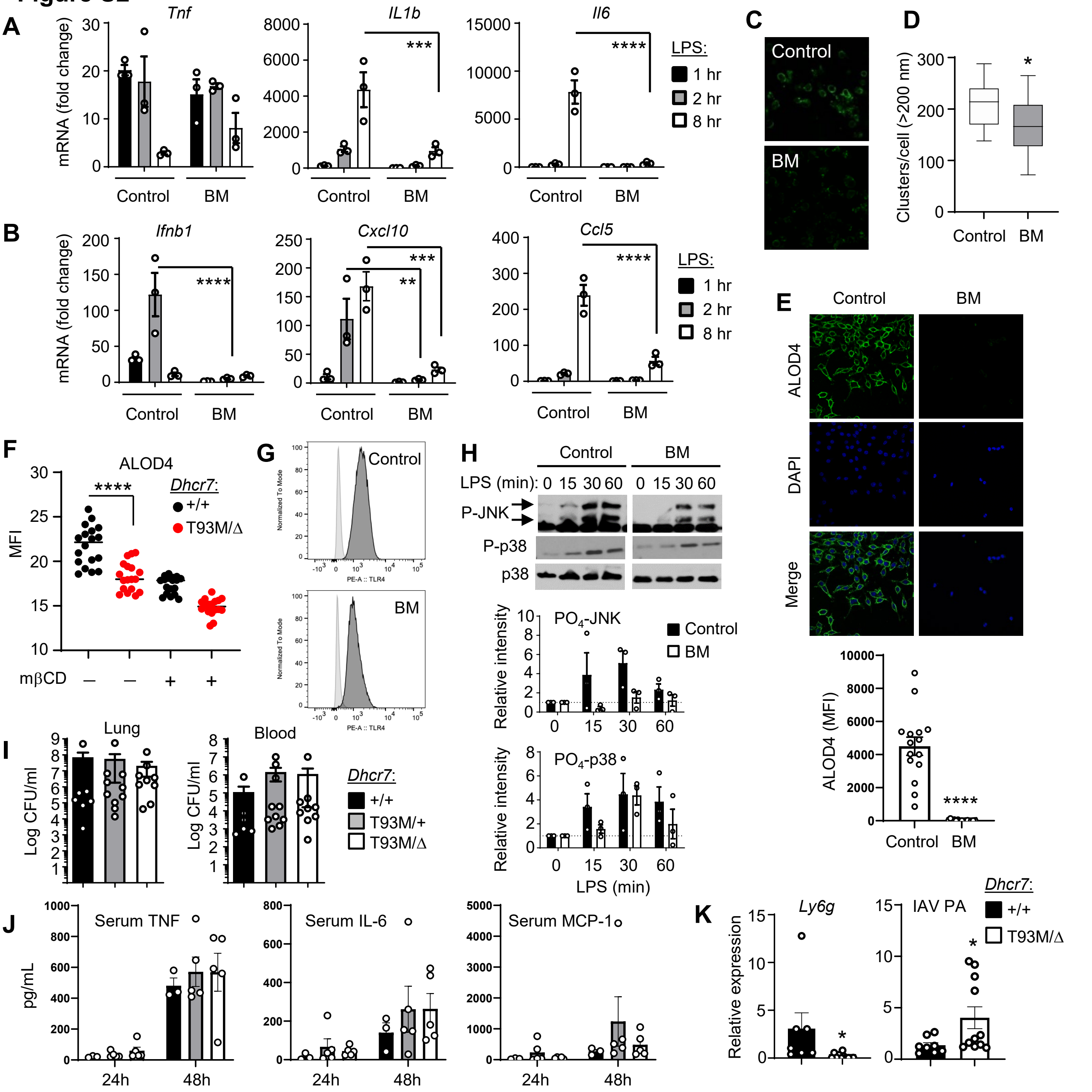

Figure S2. (**A-B**) RAW 264.7 cells were cultured in complete media with vehicle or lipoprotein-deficient media with BM15.766 (BM) for ~4 days, stimulated with LPS (1 ng/ml) for indicated durations, and evaluated by qPCR for (A) MyD88-dependent and (B) MyD88-independent cytokines. Transcripts are normalized to respective untreated controls. (**C-D**) RAW 264.7 cells were cultured as in A-B and stained with Alexa Fluor 488-cholera toxin B (CtB) and imaged (63x oil objective)(C, representative image) or analyzed by dSTORM microscopy (D) to quantify >200 nm CtB clusters. (**E**) RAW 264.7 cells cultured as in A-B were stained with ALOD4 and DAPI (nuclear stain). Representative images (top); quantitation (bottom). (**F**) Murine peritoneal macrophages were treated or not with methyl-β-cyclodextrin (mβCD; 5mM, 30 min) and ALOD4 staining quantified. (**G-H**) RAW 264.7 cells cultured as in A-B were (G) evaluated by flow cytometry (representative histogram for anti-TLR4 [dark grey], isotype control [light grey]) or (H) exposed to LPS, lysed, and immunoblotted. Densitometry (below), representative of 3 independent experiments. (**I-J**) Mice of indicated *Dhcr7* genotype (N=8-11 for I; N=3-5 for J) were infected i.t. with *K. pneumoniae* and lung/blood bacterial culture forming units (CFU) quantified 48h later (I) or serum cytokines quantified 24 and 48h later (J). p=NS. (**K**) Mice of indicated genotype (N=8-12) were infected i.t. with influenza A/PR8/34 and lung homogenate assayed by qPCR for targets shown. Data are mean ± SEM, representative of 2-3 independent experiments. In D, boxes are 25th-75th percentile around median; whiskers are minimum and maximum. Panels A-B analyzed by 2-way ANOVA with post-hoc test. In G, BM effect: $p<0.05$ for PO_4-JNK, P=NS for PO_4-p38 by 2-way ANOVA. *, $P<0.05$; **, $P < 0.01$; ***, $P<0.001$, ****, $P<0.0001$.
