# Supplementary figures and images for: Sterol biosynthesis regulates TLR signaling and the innate immune response in a Smith-Lemli-Opitz syndrome model
Source: J Clin Invest. 2024 Jan 18;134(6):e167633. doi: 10.1172/JCI167633 (PMC10940081; doi:10.1172/JCI167633)

Figure 1J

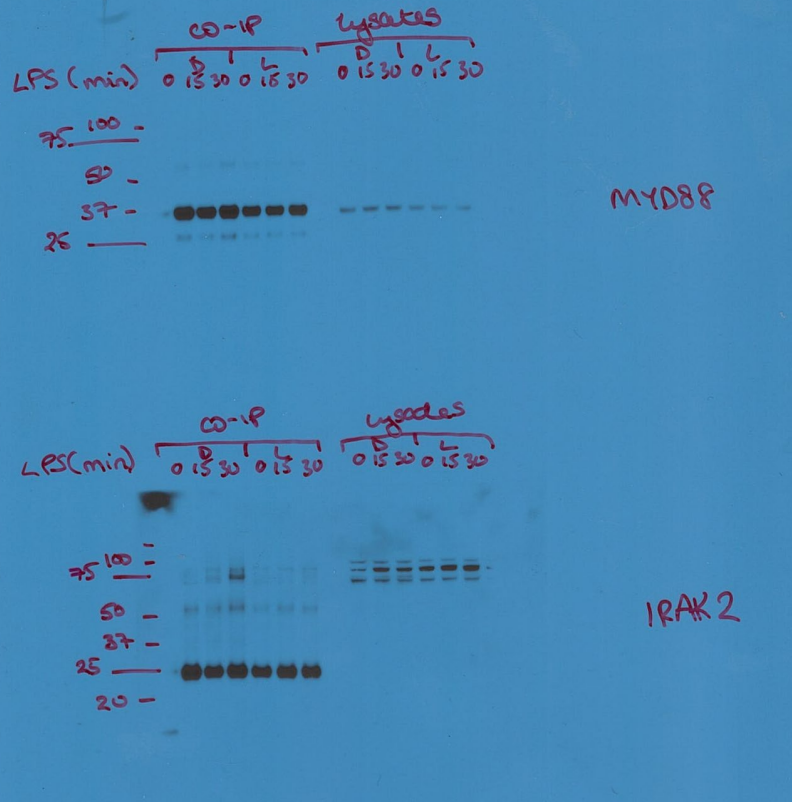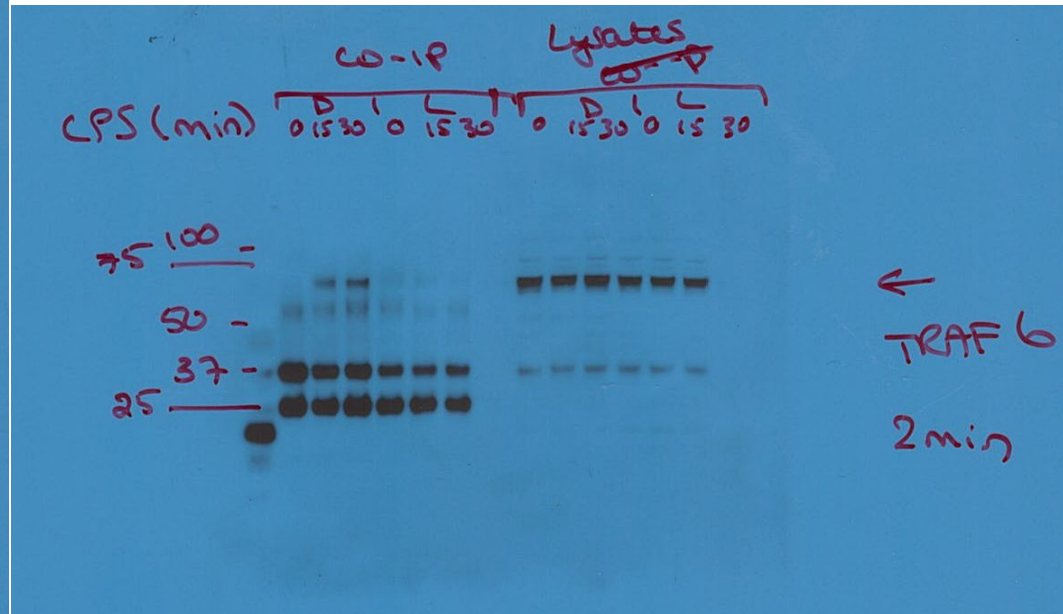

goat anti-MyD88 (R&D Systems)  
 rabbit anti-TRAF6 (Santa Cruz)  
 Rabbit anti-IRAK2 (ProSci)

Figure S2H

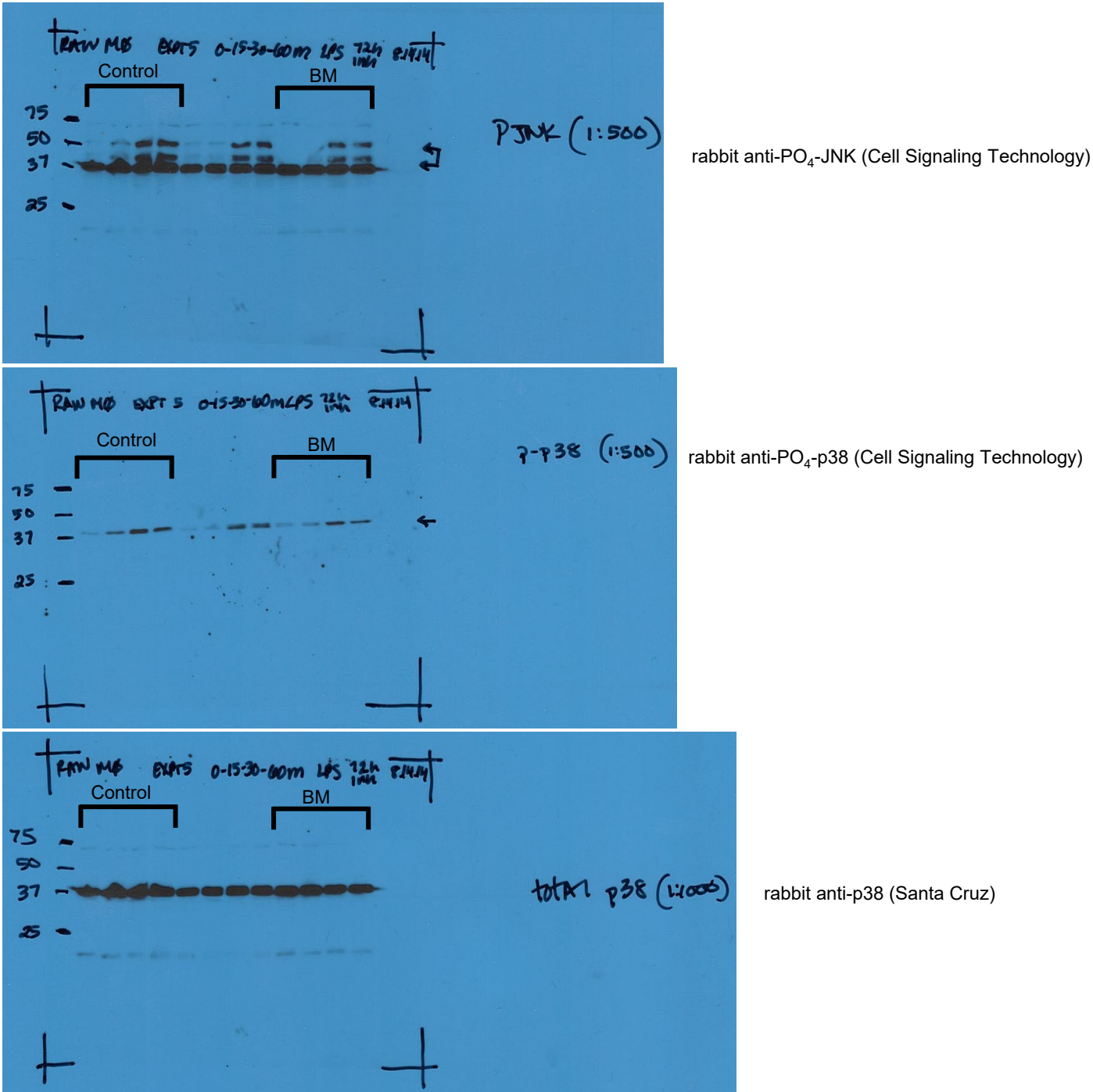

Supplement: Unedited blot and gel images [file jci-134-167633-s044.pdf]
